# Participants’ baseline characteristics and feedback of the nature-based social intervention “friends in nature” among lonely older adults in assisted living facilities in finland: a randomised controlled trial of the RECETAS EU-project

**DOI:** 10.1186/s12877-024-05408-0

**Published:** 2024-10-07

**Authors:** Kaisu H. Pitkala, Laura Rautiainen, Ulla L. Aalto, Hannu Kautiainen, Annika Kolster, Marja-Liisa Laakkonen, Timo Partonen, Hanna-Maria Roitto, Timo E. Strandberg, Nerkez Opacin, Sibylle Puntscher, Uwe Siebert, Laura Coll-Planas, Ashby L. Sachs, Jill S. Litt, Anu H. Jansson, For the RECETAS project, Acurio David, Bártová Alzbeta, Cattaneo Lucie, Holmerova Iva, Garcia Gabriela

**Affiliations:** 1 Department of General Practice and Primary Health Care, University of Helsinki, PO Box 20, 00014 Helsinki, Finland; 2 Unit of Primary Health Care, Helsinki University Hospital, Helsinki, Finland; 3 The Finnish Association for the Welfare of Older Adults, Helsinki, Finland; 4 University of Helsinki, Helsinki, Finland; 5 Department of Geriatrics, Helsinki University Hospital, Helsinki, Finland; 6 Health Services, Western Uusimaa Wellbeing Services, Espoo, Finland; 7 Department of Social Services and Health Care, Geriatric Clinic, Helsinki Hospital, City of Helsinki, Finland; 8 Department of Healthcare and Social Welfare, Finnish Institute for Health and Welfare, Helsinki, Finland; 9 University of Helsinki, and, Helsinki University Hospital , Helsinki, Finland; 10 University of Oulu, Center for Life Course Health Research, Oulu, Finland; 11 RMIT University, Melbourne, Australia; 12 Institute of Public Health, Medical Decision Making and Health Technology Assessment, Department of Public Health, Health Services Research and Health Technology Assessment, UMIT TIROL - University for Health Sciences and Technology, Hall in Tirol, Austria; 13 Institute for Technology Assessment and Department of Radiology, Massachusetts General Hospital, Harvard Medical School, Boston, MA USA; 14 Center for Health Decision Science, Departments of Epidemiology and Health Policy & Management, Harvard T.H. Chan School of Public Health, Boston, MA USA; 15 Research Group On Methodology, Methods, Models and Outcomes of Health and Social Sciences (M3O), Faculty of Health Sciences and Welfare. Centre for Health and Social Care Research (CESS), Vic, Spain; 16 University of Vic-Central University of Catalonia (UVic-UCC). Institute for Research and Innovation in Life Sciences and Health in Central Catalonia (IRIS-CC), Vic, Spain; 17 Barcelona Institute for Global Health (ISGlobal), Barcelona, Spain; 18 CIBER Epidemiología y Salud Pública (CIBERESP), Madrid, Spain

**Keywords:** Loneliness, Nature-based intervention, Health-related quality of life, Assisted living facility, Randomised controlled trial

## Abstract

**Background:**

Loneliness is common among older adults in institutional settings. It leads to adverse effects on health and wellbeing, for which nature contact with peers in turn may have positive impact. However, the effects of nature engagement among older adults have not been studied in randomised controlled trials (RCT). The “Friends in Nature” (FIN) group intervention RCT for lonely older adults in Helsinki assisted living facilities (ALFs) aims to explore the effects of peer-related nature experiences on loneliness and health-related quality of life (HRQoL). In this study we aim describe the participants’ baseline characteristics of the RCT, feasibility of FIN intervention and intervention participants’ feedback on the FIN.

**Methods:**

Lonely participants were recruited from 22 ALFs in Helsinki area, Finland, and randomised into two groups: 1) nature-based social intervention once a week for nine weeks (*n* = 162) and 2) usual care (*n* = 157). Demographics, diagnoses and medication use were retrieved from medical records, and baseline cognition, functioning, HRQoL, loneliness and psychological wellbeing were assessed. Primary trial outcomes will be participants’ loneliness (De Jong Giervald Loneliness Scale) and HRQoL (15D).

**Results:**

The mean age of participants was 83 years, 73% were female and mean Minimental State Examination of 21 points. The participants were living with multiple co-morbidities and/or disabilities. The intervention and control groups were comparable at baseline. The adherence with intervention was moderate, with a mean attendance of 6.8 out of the nine sessions. Of the participants, 14% refused, fell ill or were deceased, and therefore, participated three sessions or less. General subjective alleviation of loneliness was achieved in 57% of the intervention participants. Of the respondents, 96% would have recommended a respective group intervention to other older adults. Intervention participants appreciated their nature excursions and experiences.

**Conclusions:**

We have successfully randomised 319 lonely residents in assisted living facilities into a trial about the effects of nature experiences in a group-format. The feedback from participants was favourable. The trial will provide important information about possibilities of alleviating loneliness with peer-related nature-based experiences in frail residents.

**Trial registration:**

ClinicalTrials.gov, ID: NCT05507684. Registration 19/08/2022.

## Introduction

Loneliness has been defined as a subjective emotional state associated with unfulfilled social expectations [1]. Prevalence of loneliness is about 20–30% among home-dwelling older adults [2] and 31–100% among those living in institutional settings [3]. Loneliness has adverse effects on wellbeing, health, cognition, and it also increases mortality [4, 5]. Loneliness is also associated with an increased utilization and costs of health services [6]. Furthermore, engagement in nature-based experiences is associated with wellbeing and promotes health [7, 8]. Nature contact is associated with lower mortality [7, 9]. Direct contact with biodiverse environment seems to improve immune response [7].

Nature-based practices supporting wellbeing among residents in institutional care have predominantly included gardening and animal-assisted therapy [10, 11]. However, the associations of nature experiences with health and wellbeing have mainly been investigated in observational studies or in less-robust pre-post designs and small sample sizes [12, 13]. To our knowledge, the effects of nature experiences on loneliness have only been examined among younger people (“social prescribing”) and scarcely with controlled design [13]. Nature has been an important resource during and after the COVID-19 pandemic when older adults’ contacts were restricted and nature became a source of resilience [14].

There are studies that have explored the associations of social prescribing or other interventions on loneliness [13, 15, 16]. These studies have examined outcomes on variables such as wellbeing, loneliness and social relations. To our knowledge, only one randomised trial has shown positive effects specifically on health and mortality among community-living older adults [17]. This intervention, “Circle of Friends,” is modified to include nature experiences in the present study to examine the effects of nature-and group-based social intervention (NBSI) on lonely older adults living in assisted living facilities (ALF), herein referred to as “Friends in Nature (FIN)”.

We designed a randomised, controlled trial (RCT) with a 12-month follow-up to investigate the effectiveness of the NBSI, “Friends in Nature – Helsinki,” on older adults living in Helsinki ALFs and suffering from loneliness [18]. Our main goal of the trial is to clarify the effects of this intervention on loneliness and health-related quality of life (HRQoL) for adults living in ALF. In this article, we aim to describe the recruitment, participants’ baseline characteristics, feasibility of the intervention, and intervention participants’ feedback.

## Methods

This “Friends in Nature – Helsinki” trial is part of the RECETAS European project H2020 (Re-imagining Environments for Connection and Engagement: Testing Actions for Social Prescribing in Natural Spaces) [18, 19]. The trial was approved by the Ethics Committee of Helsinki University Central Hospital, and the procedures were planned in accordance with the Declaration of Helsinki.

### Recruitment and participants

The “Friends in Nature – Helsinki” trial design is a single-blinded RCT with two arms (intervention and control groups). The assessors of outcomes are blinded to the group allocation. The participants in the intervention arm receive NBSI intervention for nine weeks in addition to routine management in their ALF. The participants in the control group receive only the routine management at their own ALFs.

The screening with recruitment took place between August 30th, 2022 and September 5th, 2023. A total of 854 patients living permanently in ALFs in a metropolitan area of Finland were screened to take part in the study. In 2021, all Helsinki City assisted living facilities were contacted and invited to take part in the study. Of the 17 units, 12 units agreed to participate. Of those, one unit participated in the pilot study and was excluded from the main trial due to risk of contamination. When it became obvious in 2022 that there would not be a sufficient number of participants, we contacted also assisted living facilities managed by non-profit organisations and invited them to take part in the study. Of those additional 22 facilities, 13 units agreed to participate. All individuals living in these facilities and their relatives received a letter containing information on the trial. We arranged an information session in each facility for all those interested in the study. Subsequently, the facilities provided us with a list of volunteering participants having a score less than 4 in Minimum Data Set cognitive performance scale (CPS) [20]. All these residents (N = 854) were interviewed with a structured questionnaire to screen and recruit participants into the trial. Potential participants were asked: “Do you suffer from loneliness?” with options often or always/sometimes/seldom or never. Those suffering from loneliness at least sometimes were invited to the trial. At this screening interview it was ensured that recruited participants fulfilled the inclusion criteria: (1) age ≥ 55 years, (2) suffered from loneliness, (3) volunteered to participate, (4) had Mini Mental State Examination (MMSE) [21] of at least 15, (4) were able to move about at least with assistance, (5) were not deaf, (6) were able to communicate in Finnish, (7) lived permanently in an ALF, and (8) had estimated life expectancy of at least 6 months. All those having the MMSE between 15 and 18 had to have the closest proxy (legal guardian, spouse, or the closest relative) available to sign an informed consent.

**Fig. 1:**
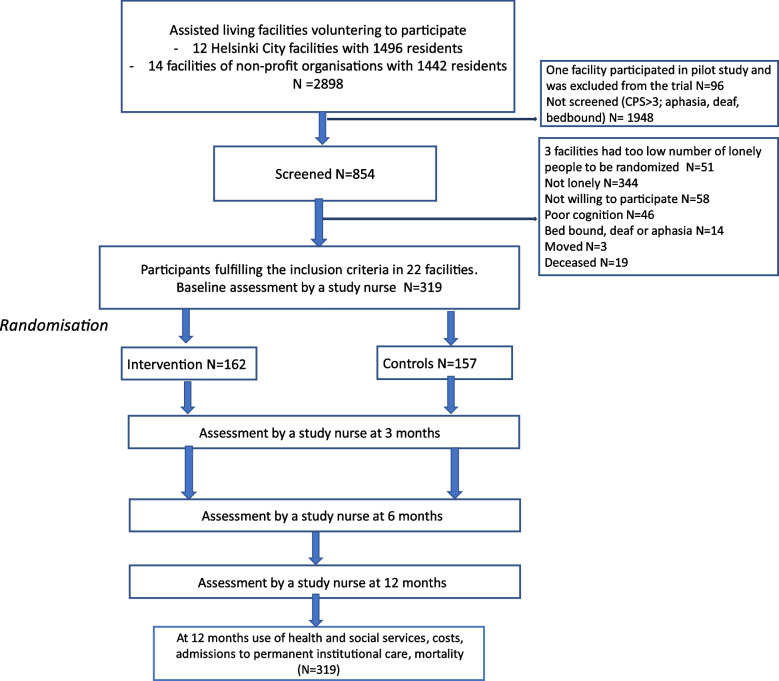
Flow chart of the study. *N* = number, CPS = Cognitive Performance Scale by Morris et al. 1994

The intervention groups were arranged in each facility, and thus, each facility was randomised separately. Therefore, we needed to recruit at least 10 individuals from each facility to form an intervention group of five participants. Three facilities had such few lonely volunteering residents that we had to exclude them. After excluding those not lonely (*n* = 344), those unwilling to participate (*n* = 58), those with poor cognition (*n* = 46), and those who were bedbound or suffering from aphasia or deafness (*n* = 14), 341 participants were eligible for the trial. By the time we started baseline assessments, 19 had been deceased and 3 moved away from the respective facility (see Fig. 1 for flow chart).

Informed consent was obtained from each participant before any study procedures. In case of a patient’s reduced judgment capacity (the MMSE score between 15 and 18), the closest proxy (spouse or relative) provided informed consent.

### Randomisation

All baseline information was collected before randomisation. Participants were randomised into the intervention group (*n* = 162) or control group (*n* = 157) using computer-generated randomly allocated numbers received by telephone from a randomisation centre. To enable intervention groups including 5 to 8 participants, the residents were randomised separately in each facility. This enabled the groups to meet together easily in their own facility and gave possibility for the groups to continue their meetings after the official group process had been completed.

### Measurements

Two registered nurses assessed the participants at four meetings: at baseline as well as at 3, 6 and 12 months from baseline. The baseline assessments were performed between February 6th, 2023 and September 28th, 2023. The demographic data were confirmed from medical records. Education and marital status were inquired and confirmed from proxy when necessary. Diagnoses and medications were retrieved from medical records and Charlson comorbidity index was calculated to measure the severity of disease burden [22]. Self-rated health was assessed by a single question “In general, how would you rate your health today” with four answer choices (very good, good, fair, or poor) and divided as good (very good or good) or poor (fair or poor). Quality of sleep was inquired by a single question [23] “Do you think you get enough sleep? (yes, almost always/yes, often/seldom or hardly ever/do not know) and divided as getting enough sleep (almost always or often) or not (hardly ever or do not know).

The dementia severity and cognitive status of participants were assessed at baseline using Clinical Dementia Rating (CDR) [24] and Mini Mental State Examination (MMSE) [21], respectively. Executive function was further evaluated using Clock Drawing Test [25] and semantic memory by Verbal Fluency Test [20].

Physical functioning was assessed using Barthel Index [26], and frailty was evaluated by frailty phenotype [27]. Mobility was inquired by a single item “Does your general health allow you to walk easily outdoors? (Yes/No, I need an assistive device/No, I need help of another person/No, I cannot walk, I need wheelchair outdoors). The item was divided (Independent: “Yes” / Independent with an assistive device: “No, I need an assistive device” / Only with another person’s help: “No, I need help of another person” or “No, I cannot walk, I need wheelchair outdoors”). Depression at baseline was measured by Geriatric Depression Scale (GDS) [28].

The *primary outcome* measures of the trial are the 11-item De Jong Gierveld Loneliness scale (DJGLS) [29], and the 15-dimensional instrument (15D) to assess HRQoL [30]. The standardized De Jong Gierveld Loneliness scale measures both social and emotional loneliness [29, 31]. The maximum score is 11 points, with 0 to 2 points identifying no loneliness and 3 to 8 points indicates moderate loneliness, 9 to 10 severe loneliness and 11 very severe loneliness. DJGLS can also be divided into social loneliness score (max. 5 points) and emotional loneliness score (max. 6 points). 15D can be administered in an interview with the subject or his/her proxy. 15D is a standardized generic questionnaire, which includes 15 multiple-choice items to measure, for example, on mobility, sleeping, eating, usual activities, mental function, discomfort and symptoms, depression, and vitality [30]. It can be used both as a profile measure and a single index score measure varying between 0 (poor HRQoL) and 1 (excellent HRQoL) [30]. The 15D is sensitive to change after a health care intervention [32]. 15D correlates well with other HRQoL measures such as SF-20 or EQ-5D [30].

Secondary outcomes include (1) psychological wellbeing according to the Psychological Wellbeing (PWB) scale [33], (2) cognitive tests: Clock Drawing, and Verbal fluency, [20, 25], (3) quality of sleep [23], and (4) frailty status according to frailty phenotype [27]. PWB includes six questions: 1. life satisfaction, 2. feeling needed, 3. having plans for the future, and 4. zest for life (all yes/no); as well as 5. feeling oneself depressed, and 6. suffering from loneliness (alternatives: often or always/sometimes/seldom or never) [32]. These simple questions have been used among people with dementia and found easy to understand and respond. We created a wellbeing score, where each question represented 0 (“no” in questions 1 to 4, “often or always” in questions 5 or 6), 0.5 (“sometimes” in questions 5 or 6) or 1 (“yes” in questions 1 to 4, “seldom or never” in questions 5 or 6) point. The score is calculated by dividing the total points by the number of questions the participant had answered. Thus, a score of 1 represents the best and 0 the poorest wellbeing. The score shows good concurrent validity with RAND-36 [33].

This study is single blinded. Experienced study nurses are blinded to group assignment and conduct all the assessments. Participants know when they are receiving intervention but they do not know the outcome measures. Additionally, data on use and costs of health and social services will be gathered from hospital and social service documents and medical records, and dates of death from the central registers until 12 months from baseline by researchers unaware of the group allocation.

### Intervention

Altogether 52 facilitators were trained for the “Friends in Nature” groups. The model of this nature-based social group intervention and facilitator training were modified from Circle of Friends model [34, 35]. There were two or three professionals from each ALF. The original Circle of Friends facilitator training has been described elsewhere [35].

Before the groups started, each participant allocated to the intervention group was interviewed individually by two facilitators from the same ALF. After the interview, each participant received a personal empowerment letter emphasizing her/his strengths and supporting participation in intervention. Participants in the intervention groups met at their own ALF once a week nine times altogether. Each group consisted of five to eight older participants and two professional group facilitators. Duration of each meeting was 1.5–2 h, having objective-oriented group program [34–36] that the participants could modify.

Everything was free of charge for the participants. The contents of the groups were related to loneliness and nature. The process and principles of group intervention were similar in all groups. The professional facilitators aimed to enhance security and equal communication in the groups. They empowered older participants and promoted friendships between them by taking advantage of group dynamics and the normal maturation of a group process [36]. All participants were interested in nature. Therefore, the intervention encouraged the participants to share their experiences with peers of their own age and same interests, discuss their feelings of loneliness, and receive peer support. This in turn was aimed to lead to empowerment of the participants and better self-efficacy. Towards the end of group process the facilitators were to step back and give power to older participants in the groups, so that they would continue to meet their group members on their own after the facilitated group process was completed.

### Assessing feasibility and process of intervention

The facilitators wrote field diaries on each group session. In these half-structured diaries, they took notes about the daily weather, who was present, and reasons for nonattendances. They also documented what the group had done during the sessions: excursions, discussions, atmosphere, group roles, humour, and quarrels. We could retrieve information on compliance, excursions, and weather conditions from these diaries.

The participants filled in a questionnaire after the group intervention was over. It included questions (yes/no) concerning how the participants had felt about the group intervention.

### Statistical analysis

Sample size for the “Friends in Nature – Helsinki” trial was calculated based on the primary outcome measure 15D [18]. The calculation was based on a typical standard deviation (SD) in this type of population 0.11, type I error 5% and power 80%. We estimate a loss to follow-up of 25%. As the clinically significant difference between the intervention and control arms is 0.04 [30], the number of participants per randomisation arm was computed to be 158, yielding a total sample size of 316.

Baseline statistical analyses included standard descriptive statistics of the participants, that is, group means with standard deviation (SD), or absolute numbers with percentages (%). The differences between the intervention and control groups were analysed using chi square-test, Fisher’s exact test, Mann–Whitney *U* test or *t*-test for independent samples as appropriate. The level of statistical significance was set at 0.05 for all analyses. The feedback data are presented as percentages.

## Results

After screening for the inclusion criteria, a total of 319 residents were assessed between February 6th and September 28th, 2023 in ALFs and were included in the trial. Randomisation is shown in Fig. 1. The baseline assessments were performed between February and September 2023 whereafter the participants were randomised into intervention and control arms. The intervention participants’ feedback questionnaires were completed immediately after the group intervention was over, the last one in December 2023.

### Baseline findings

There were no significant differences in demographic characteristics, health status or wellbeing between the persons randomised into the intervention and control groups (Table 1). The mean age of the 319 participants was 83 years, and a higher proportion were females (73%). The participants had a high number of comorbidities and more than half of them had a diagnosis of dementia. However, the mean MMSE was 21 (Table 1).

**Table 1:** Baseline characteristics of participants

	Control (*n* = 157)	Intervention (*n* = 162)	*P*-value
*Demographics*			
Women, n (%)	117 (75)	115 (71)	0.48
Age in years, mean (SD^a^)	83 (8)	83 (9)	0.87
Education, n (%)			0.17
Primary school or less	50 (32)	63 (39)	
Middle	91 (58)	77 (47)	
University	16 (10)	22 (14)	
Marital status, n (%)			0.53
Married	28 (18)	36 (23)	
Unmarried or separate	66 (43)	60 (38)	
Widowed	59 (39)	62 (39)	
*Health*			
Hears normal speech, n (%)	148 (95)	154 (97)	0.53
Charlson Comorbidity Index^b^, mean (SD)	1.9 (1.3)	2.0 (1.4)	0.66
Dementia^c^, n (%)	93 (59)	84 (52)	0.18
Stroke^c^, n (%)	36 (23)	40 (25)	0.71
Number of medications, mean (SD)	9.8 (3.6)	9.2 (3.8)	0.12
Self-rated health good, n (%)	125 (78)	127 (78)	0.79
Sleeps enough, n (%)	129 (82)	134 (83)	0.90
*Physical functioning*			
Mobility while outside, n (%)			0.80
Independent without a device	32 (20)	38 (24)	
Independent with a cane or rollator	66 (42)	66 (41)	
Needs another person’s help	59 (38)	58 (36)	
Barthel Index^d^, mean (SD)	63(28)	65 (27)	0.61
Frailty^e^ n (%)			
Robust	2 (1)	0 (0)	0.30
Prefrail	49 (31)	56 (35)	
Frail	106 (68)	106 (65)	
*Cognition*			
MMSE^f^, mean (SD)	21.3 (4.6)	21.0 (5.0)	0.60
CDR^g^			0.69
0–0.5, n (%)	50 (32)	55 (34)	
1–2, n (%)	107(68)	107 (67)	
CDT^h^, mean (SD)	2.4 (2.1)	2.4 (2.1)	0.93
VF^i^, mean (SD)	10.7 (5.2)	10.7 (5.5)	0.65
*Psychological wellbeing*			
15D^j^, mean (SD)	0.696 (0.092)	0.701 (0.088)	0.62
DJGLS^k^ total, mean (SD)	5.8 (2.8)	5.8 (2.7)	0.82
Social, mean (SD)	2.2 (1.8)	2.3 (1.7)	0.60
Emotional, mean (SD)	3.6 (1.8)	3.5 (1.7)	0.42
Number of close people, mean (SD)	3.1 (2.4)	3.4 (4.2)	0.78
GDS^l^, mean (SD)	5.2 (3.3)	5.1 (3.0)	0.70
PWB^m^, mean (SD)	0.57 (0.25)	0.60 (0.21)	0.27

*n* number

^a^*SD *standard deviation

^b^Charlson et al. 1987

^c^Active diagnoses in medical records

^d^Mahoney and Barthel 1965

^e^Frailty phenotype by Fried et al. 2001

^f^Mini Mental State Examination by Folstein et al. 1975

^g^*CDR *Clinical Dementia Rating by Hughes et al. 1982

^h^*CDT *Clock Drawing Test by Forti et al. 2010

^i^Verbal Fluency by Morris et al. 1994

^j^15D 15-dimentional health related quality of life by Sintonen 2001

^k^*DJGLS *De Jong Gierveld Loneliness Scale by De Jong Gierveld and Van Tilburg 2006, 2010

^l^*GDS *Geriatric Depression Scale by Kurlowicz,& Greenberg 2007

^m^*PWB *Psychological Wellbeing Scale by Routasalo et al. 2009

While the mean Barthel Index was 64 among the participants, only 22% were able to move independently outdoors without assistive devices. Because nearly all participants needed a wheelchair to visit nature sites, this presented challenges to the group excursions. Therefore, nearly all participants needed wheelchair accessible taxies to transfer them to the nature destinations. Two in three were frail, according to their frailty phenotype.

The mean DJGLS was 5.8 (SD 2.8), indicating moderate loneliness at baseline. The mean 15D was 0.699 (SD 0.090). The mean GDS was 5.1 (SD 3.1), indicating that some participants had possible mild depression. Mean PWB was 0.59 (SD 0.23). The participants had a mean 3.2 close people (either relatives or friends).

In general, comparison of the intervention and control groups indicated that randomisation was successful. The randomised groups did not differ significantly in any of the baseline characteristics assessed.

### Feasibility of intervention

Altogether 22 ALFs with 25 groups and in total 162 people were randomised into the intervention arm. The groups made a mean 3.6 (range 2–6) excursions to nature. There was a wide range of places visited, reflecting that the participants had a possibility to choose what they wanted. However, most of the group sessions were performed indoors and had nature as an activity and discussion topic. The weather in Helsinki metropolitan area was not optimal most of time for nature excursions. The mean temperature outside during the group sessions was 8.5 °C. Of the 235 group session days, sessions were held under a variety of weather conditions: partial sun (48%), partial clouds (42%), rain (14%), and snow and icy conditions (12%) and windy days (22%).

Compliance with intervention was only moderate, with a mean attendance of 6.8 (76%) out of the 9 sessions. Altogether 16 participants refused to participate or participated at less than three sessions. Further seven participants fell ill or were deceased during the group process, and they could participate only maximum three sessions. A total of 55 participants attended all nine sessions. Among all scheduled sessions, there were 55 nonattendances due to illnesses, 21 due to quarantines (COVID-19 or Norovirus), and 74 due to other reasons (a doctor’s visit, having a relative to visit, forgetting, or being too tired).

We received quite favourable feedback from those participating in the groups. Of the intervention group participants, 136 responded to the feedback questionnaire (response rate 83%). The answers to this feedback questionnaire are summarized in Table 2. About 57% of the respondents reported that the group had alleviated loneliness, and 80% stated that loneliness was understood in the group. However, only 25% had made new friends in the group and only 3 out of the 25 groups continued the group meetings on their own after the facilitated group process was over. The respondents appreciated the facilitators. Of the respondents, 96% would have recommended a respective group to other older adults. Most participants did not consider the group sessions too heavy (81%) or exhaustive (79%).

**Table 2:** Participants’ feedback on group intervention

Question (yes/no)	Yes (%)
Did you feel that your loneliness was alleviated?	57
Were the topics of group discussions important to you?	73
Did you experience that the feelings of loneliness were understood in the group?	80
Have you made friends with someone in the group?	27
Do you keep contact with someone in the group?	15
Has your group had a meeting after the official group sessions were over?	3/25 groups
Did you receive peer support in your group?	60
Did you have a possibility to influence the group program?	60
Was the program in the group in line with your wishes?	73
Did the group facilitators have competence to facilitate your group?	96
Did the group facilitators value you?	97
Would you join a respective group again?	84
Would you recommend “Friends in Nature” group to other older people?	96
Did you have nature experiences in the group?	84
Was the staff in your assisted living facility supportive to the group meetings?	88
What did the group meetings mean to you?	
Meeting other people?	93
Being able to go to the nature?	89
To experience new things?	62
Joy of waiting?	70
Enjoying nature?	93
Exhaustion after the day?	21
Heavy days?	19

Numerous participants provided responses to open-ended questions as well. There was plenty of feedback about how nice it was to go on excursions, meet and get acquainted with new people, and be in a group with a good spirit. The participants gave feedback that there were too little discussions on loneliness. They also criticised that their group was cognitively heterogeneous, and that some group members had poor memory. Some were disappointed about their own condition, which did not allow them to enjoy enough of nature. Some said that 9 sessions was insufficient to form real friendships. Several participants gave feedback that they would like to have continued their meetings. However, most said that there was nothing to criticize in the group meetings.

## Discussion

We have successfully randomised 319 lonely residents from assisted living facilities, aged 55 years and older, into two arms to investigate the effects of a 9-week nature-based intervention performed in their own facilities in the Helsinki metropolitan area, Finland. The baseline data is complete, and the randomised groups are well-balanced. Participants in the intervention and control groups did not differ significantly in any of their characteristics. Furthermore, primary outcome measures loneliness and HRQoL were similar in both groups at baseline. Based on session adherence and participant feedback, the “Friends in Nature – Helsinki” intervention was only partly accepted. Of the intervention group participants, 14% attended three or less of their group meetings due to either refusals, being ill or quarantined for COVID-19 or Norovirus epidemics. In our previous trial targeted on home-welling lonely older people, only 2.5% dropped out from the intervention group [17]. Of the respondents, 57% reported a subjective benefit of intervention on loneliness, but 96% would recommend the group intervention to other older adults. Due to the Northern latitude and potentially challenging weather conditions in Finland, it was sometimes difficult to arrange nature excursions for frail older group participants.

The characteristics of the participants in our trial are comparable to respective populations in ALFs [37, 38]. The participants in our trial are older, frail and have a high number of comorbidities and disabilities. Due to our inclusion criteria, although cognition was better in our participants than those in previous studies [37, 38], our participants were more often frail. The HRQoL was higher than in a previous study [39], suggesting that selecting residents with better cognitive skills has impact on their functioning and overall HRQoL. However, due to loneliness, their PWB was lower than a respective ALF population in a previous study [38].

Due to the frailty, multimorbidity and disabilities in the population, we had to modify our intervention. The original “Circle of Friends” intervention had been shown to improve HRQoL, wellbeing and health and to reduce mortality [17]. In our current trial, we had to shorten the sessions from 6 to 2 h. In our pilot study it became obvious that our participants were not able to attend such long days. Moreover, “Friends in Nature” intervention consisted of 9 sessions instead of 12 sessions in original “Circle of Friends.” However, the ideas of empowerment, using group dynamics and peer support to enhance self-efficacy were included in the intervention of the present trial. The contents of group sessions were related to nature with excursions to nature. The challenges due to weather conditions were noted above.

The intervention received favourable feedback from the participants. About 57% of the respondents in the intervention group reported that the group had alleviated loneliness. However, this fraction was much lower than in our previous studies, in which 87% stated that their loneliness had been alleviated [5]. Furthermore, only the DJGLS will eventually provide us the information whether the intervention is truly effective on loneliness when the intervention and control groups will be compared during the 12 months follow-up. In our previous follow-up study, about 60% of the community-living Circle of Friends groups continued after the intervention on their own [35], whereas in the present study; the only 12% (3/25) of the FIN groups continued on their own. The residents in ALFs need support from nurses. For example, they need functional aids, reminders due to impaired cognition, and support to socialise with others. This is probably a challenge in the facilities, with their hurried working days. Also, the high assistance needs requires that accessible natural spaces are close to ALFs. Nevertheless, it was reassuring and important that 96% would recommend these groups to other residents. The feedback in the open-ended questions echoed this. Nearly all participants appreciated the nature trips and getting acquainted with new people. However, it seems that some groups were too heterogeneous due to varying cognitive capacities. Some participants also considered the group process too short and this may have influenced the intervention effects.

Our trial has both strengths and limitations. A systematic, theoretically coherent “Friends in Nature” intervention was designed to fit our study population. The primary and secondary outcomes are validly measured, and HRQoL is a clinically meaningful outcome. We had a fairly large number of dropouts. However, this has already been considered in our sample size calculation, and our sample should nevertheless be large enough to show effects if there are any. Experienced study nurses ensure the validity of data collection, and all the assessors are blinded to group assignment.

In order to maximize external validity and transportability to other populations, the exclusion criteria of our trial were kept to a minimum. We allowed both mild and moderate stages of dementia, as well as very old persons with comorbid conditions. This heterogeneity, including disabilities, comorbidities and cognitive decline of the participants, may dilute our findings. Randomisation stratified by ALF was challenging, and some ALFs had to be dropped due to low number of participants.

## Conclusions

We designed and initiated a large RCT investigating the effects of a nature-based and group-based social intervention on lonely older residents living in ALFs with the aim to improve the main outcomes HRQoL and loneliness and the secondary outcomes wellbeing and cognition. Baseline assessments were performed, randomisation was successful, and feedback of the intervention participants was quite favourable. The “Friends in Nature – Helsinki” trial will provide important information on the effects of nature and group-based social intervention among frail, lonely, older adults living in ALFs.

## Data Availability

The datasets generated and analysed during the current study are not publicly available due but are available from the corresponding author on reasonable request.

## Abbreviations

15D: 15-Dimensional instrument
ALF: Assisted living facility
CDR: Clinical Dementia Rating
CDT: Clock Drawing Test
CPS: Cognitive performance scale
DJGLS: De Jong Gierveld Loneliness scale
FIN: Friends in Nature
GDS: Geriatric Depression Scale
HRQoL: Health-related quality-of-life
MMSE: Mini-Mental State Examination
NBSI: Nature-based social intervention
PWB: Psychological Wellbeing
RCT: Randomised, controlled trial
RECETAS: Re-imagining Environments for Connection and Engagement: Testing Actions for Social Prescribing in Natural Spaces
SD: Standard deviation
VF: Verbal Fluency
